# An interactive assistant for patients with cardiac implantable electronic devices: A study protocol of the LUCY trial

**DOI:** 10.1097/MD.0000000000012556

**Published:** 2018-09-28

**Authors:** Joanna Michalik, Andrzej Cacko, Jakub Poliński, Kacper Pawlik, Emanuel Tataj, Monika Gawałko, Grzegorz Opolski, Marcin Grabowski

**Affiliations:** a Department of Medical Informatics and Telemedicine; b 1st Department of Cardiology, Medical University of Warsaw, Warsaw, Poland.

**Keywords:** cardiac implantable electronic device, cardiac resynchronization therapy, cardiovascular diseases, heart diseases, heart failure, implantable cardioverter-defibrillator, mobile application

## Abstract

Patients with chronic heart failure (CHF) and reduced left ventricle ejection fraction benefit from cardiac resynchronization therapy (CRT) and implantable cardioverter defibrillator (ICD). However, increasing numbers of patient with CRT and ICD devices produce overload of cardiology centers where patients are admitted to ambulatory visits. This study aims to find multivariate model predicting the requirement for ambulatory follow-up of cardiac implantable electronic devices (CIEDs).

The LUCY study is an observational, cohort, prospective, 2-stage trial. As equal number of patients (300) will be included in the first and the second part of the study, finally, 600 patients will be included in the study. The inclusion criteria will be: age between 18 and 90 years, CHF (New York Heart Association classes I–III) and implanted ICD or CRT at least 30 days before study inclusion. The exclusion criteria will be dementia and other conditions impeding cooperation during the study. All patients included in the study will undergo standard ambulatory visit. Primary endpoint will be defined as any ambulatory visit qualified as necessary due to patient's condition or device malfunction diagnose by the cardiologist: any change in pharmacotherapy related to patient's clinical status assessed during the visit, any change in tachyarrythmia counter or discriminator status, any change in tachyarrythmia threshold, presence of ventricular undersensing or oversensing, presence of atrial or ventricular ineffective pacing, or device's pocket infection. Secondary endpoint will be defined as any ambulatory visit qualified as necessary due to the alarm identified via Medtronic CareLink Express (MCLE): sustained or treated ventricular tachyarrythmia, any not previously diagnosed supraventricular tachyarrythmia, or elective replacement indicator.

Our study is the first attempt of implementation of the machine learning and elements artificial intelligence in health care optimization of patients with CIED. The LUCY will be an open product, available for additional testing and improvement with supplementary functionalities: quality of life assessment, teleconsultation, video-streaming, automated imagine recognizing.

## 1 Introduction

Patients with chronic heart failure (CHF) and reduced left ventricle ejection fraction benefit from implantable cardioverter defibrillator (ICD).^[1]^ Among them, individuals with electrocardiographic signs of left ventricle contraction asynchrony are candidates for cardiac resynchronization therapy (CRT).^[2]^

Patients with cardiac implanted electronic devices (CIEDs), both ICD and CRT, require long-term observation. Most of them, especially in Europe, are regularly monitored during standard ambulatory visit at least twice a year.^[3]^ However, increasing numbers of patients with CRT and ICD devices lead to overload of referral cardiology centers. According to the latest publications most standard ambulatory visits are unnecessary: do not provide any additional information nor result in changes in pharmacologic treatment or significant reprogramming of the device.^[4]^ Moreover, high number of ambulatory visits reduces time and sources necessary to provide better therapy for the most complicated subjects.^[5,6]^

Currently used devices, besides cardiac pacing and delivering low- and high-energy antitachyarrythmia therapies, record patient's heart rate, activity, and many other parameters directly related to patients's hemodynamics. Whereas there are technologic possibilities for remote monitoring proven to be effective in identifying the device's damage and high risk of CHF exacerbation remote monitoring can be a solution for overload health care providers to optimize patients’ management and therapy.^[5–8]^ On the contrary, currently used remote monitoring services provide an “information noise” that is tremendous volume of data about device, involve medical staff's continuous attention, and limit clinical information.^[9]^ For this reason, an additional contact with patient for clinical information is essential to fully assess patient's health status.^[3,5,6]^ Therefore, we shall provide a new system of remote monitoring available to integrate data from implanted devices and health status reported by patient.

The aim of the study is to develop an interactive patient's assistant called LUCY and a multivariate model predicting the requirement of ambulatory follow-up of patients with ICD or CRT device.

## 2 Methods

### 2.1 Trial design

The LUCY study is an observational, cohort, prospective, 2-stage trial.

Designed in the study interactive patient's assistant, also called LUCY will be a mobile application, a database and a machine learning system operating as a data integrator. During the first stage of the study LUCY will be initiated. The relevant parameters for patient's endpoints prediction will be collected from data from remote device's interrogation via Medtronic CareLink Express (MCLE) system and health status reported by patients. Implemented real-time self-learning algorithm will predict the necessity of ambulatory visit. In the second stage of the study, the effectiveness of LUCY's prediction of patient's endpoints will be assessed.

### 2.2 Ethics and registration

This protocol has been reviewed and approved by the Institutional Review Board of the Medical University of Warsaw, number AKBE/233/2017. The protocol identification number at ClinicaTrials.gov is NCT03474315. The study will be conducted following the principles of the Declaration of Helsinki. Data monitoring committee is not planned in the study as it is an observational study.

### 2.3 Study population

As equal number of patients (300) will be included in the first and the second part of the study, finally, 600 patients will be included in the study.

Participants will be recruited among patients who are under the outpatient care of reference cardiology center and sign informed consent. The inclusion criteria are: age between 18 and 90 years, CHF (New York Heart Association classes I–III) and implanted Medtronic (Minneapolis, MN) ICD or CRT at least 30 days before study inclusion. The exclusion criteria are dementia and other conditions impeding cooperation during the study.

All patients will undergo remote control using MCLE system and record patient-reported clinical outcomes just before the ambulatory visit. Patients reported data will be collected just after remote device interrogation with a mobile device through the dedicated module of the LUCY.

**Figure 1:**
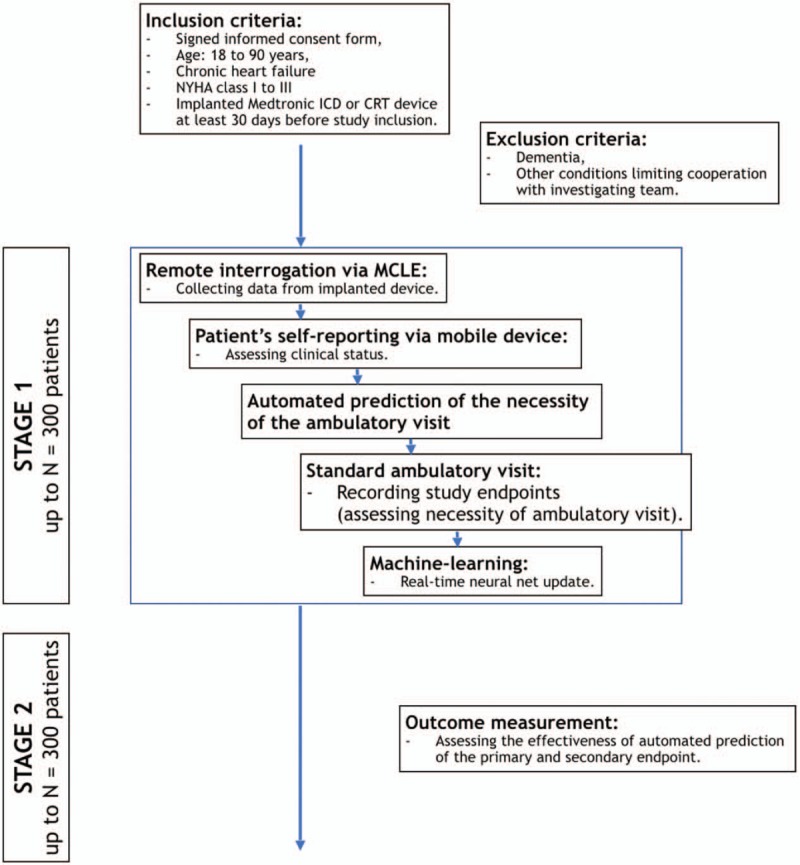
Study protocol. CRT = cardiac resynchronization therapy, ICD = implantable cardioverter-defibrillator, MCLE = Medtronic CareLink Express, NYHA = New York Heart Association.

All patients included in the study will undergo standard ambulatory visit in the referral outpatient clinic immediately after evaluation by LUCY. Every patient will be examined by an expert cardiologist blinded for result of LUCY's evaluation. Due to patient's physical examination and device interrogation cardiologist may indicate device reprogramming, changes in medications or patient's hospitalization. Cardiologist's decisions will be referral to results of LUCY's evaluation and will be considered as real-time data for machine-learning. Study protocol is presented in Figure 1.

During the study, every LUCY's module will be optimized and maintained by an interdisciplinary team to perform data acquisition and real-time implementation of automated algorithm modification to predict unnecessary ambulatory visit.

### 2.4 Study endpoints

Primary endpoint was defined as any ambulatory visit qualified as necessary due to patient's condition or device malfunction diagnose by the cardiologist: any change in pharmacotherapy (modification of agents or doses) related to patient's clinical status assessed during the visit, any change in tachyarrythmia counter or discriminator status, any change in tachyarrythmia threshold, presence of ventricular undersensing or oversensing, presence of atrial or ventricular ineffective pacing, or device's pocket infection.

Secondary endpoint was defined as any ambulatory visit qualified as necessary due to the alarm identified via MCLE: sustained or treated ventricular tachyarrythmia, any not previously diagnosed supraventricular tachyarrythmia, or elective replacement indicator.

### 2.5 Outcome assessment

The outcome of the study is a true positive result of the LUCY's estimation in predicting primary and secondary endpoint in the second stage of the trial.

#### 2.5.1 Data collection tools

##### 2.5.1.1 Medtronic CareLink Express

The effectiveness of MCLE system was previously assessed and results were published.^[4]^ All participants will be trained in using MLCE system. Device remote interrogation will be performed just before ambulatory visit via MCLE station located at the ambulatory clinic. Data collected via MCLE include: supraventricular- and ventricular-tachyarythmia episodes, ICD therapies, signal amplitudes, percentage of paced rhythm, patient's activity, thoracic impedance and fluid status, heart rate variability, mean heart rate at night and day, and battery status.

##### 2.5.1.2 LUCY's module for patient self-reported clinical status

Important module of the LUCY platform will be a tool for patients’ health status self-evaluation. It was designed as an interactive tool compiling proprietary questionnaires exploring different domains quality of life, factors affecting health (especially CHF) status, medical adherence, and device pocket status.

The data will be inputted using a tablet and dedicated web page. It should be emphasized that the group of patients with CIED is diverse: people between the ages of 18 and 90 years, for various society groups, which additional comorbidities or disabilities. The web page is very user-friendly and adjusted for people with vision defects, allowing assessment expressed on a scoring scale.

The very first patients included into the study will be asked to fulfill questionnaires validated for the Polish population:

- the World Health Organization Quality of Life Test - BREF to assess the quality of life of people under 65 years of age and adequately the World Health Organization Quality of Life Test - AGE for older people,^[10]^
- the Minimum European Health Module, a set of 3 general questions characterizing 3 different concepts of health: self-perceived health, chronic morbidity, and activity limitations,^[11]^
- the Beck depression inventory for people under 65^[12]^ and the geriatric depression scale for older people,^[13]^
- the adherence in chronic diseases scale to assess the adherence to prescribed pharmacologic treatment,^[14]^
- authors’ questionnaire of CHF symptoms exacerbation, and
- queries about device pocket's clinical status.

While the time needed to report patient's clinical status is a key issue, the neuronal net will be focused on limiting number of queries necessary to assess patient's clinical status. Limiting queries with every iteration requests implementation of an additional condition into machine-learning schema and interaction between user and built-in assessment center after every patient's answer.

### 2.6 Machine learning

Data collected from the first 300 patients will be analyzed with neural net created by interdisciplinary team of medical and technology departments. Machine learning will be performed using the R programming language (v. 3.4.3). Afterwards, the neural net will be updated real-time after every patient's assessment to predict primary and secondary endpoint with the highest effectiveness.

### 2.7 Statistical methods

#### 2.7.1 Sample size calculation

During the survey planning, we calculated the sample size required to assess the correlation between input data and primary outcome as 311, due to results of previously performed and published study (alpha = 0.01, study power = 0.95, the odds ratio for outcome indication = 3.3, outcome prevalence = 30%).^[4]^ We used G∗Power software 3.0.1.0 downloaded from http://www.gpower.hhu.de [S1]. The final number of subjects planned to input into the study is a double of calculated size (one for each stage of the trial).

#### 2.7.2 Statistical analysis

All the data will be analyzed in R (v. 3.4.3). Continuous variables will be presented as means and standard deviation while categorical variables as number and percent of subjects. For categorical variables, differences between patients will be analyzed using Pearson Chi-squared test. For continuous variables, the Kolmogorov–Smirnov test will be applied to check the distribution of data. In the case of normally distributed data independent *t* test will be used to compare patients, whereas Mann–Whitney *U* test will be used for non-normal distributions. Effectiveness of analyzed parameters and composite parameters in indicating the patient's fulfilling primary and secondary outcomes will be assessed using logistic regression. For all analysis, *P*-value <.05 will be considered significant.

## 3 Discussion

The currently available data suggest that remote monitoring in addition to standard ambulatory visit improves prognosis of patient's with CIED.^[3,5]^ However, huge amount of data generated by remote monitoring system and lack of contact with patient in the era of patient-oriented medicine strongly limits a common use of remote monitoring.

Promising applications of data mining and machine learning techniques in health-measurement algorithms allow us to assume probable benefits from using modern methods of data analysis in assessment of the required ambulatory follow-up based on data gathered through remote monitoring.

## 4 Conclusion

Our study is the first attempt of implementation of the machine learning and elements artificial intelligence in health care optimization of patients with CIED. LUCY will be an open product, available for additional testing and improvement with supplementary functionalities: quality of life assessment, teleconsultation, video-streaming, automated imagine recognizing.

## Author contributions

Conceptualization: Joanna Michalik, Andrzej Cacko, Jakub Poliński, Kacper Pawlik, Emanuel Tataj, Monika Gawałko, Grzegorz Opolski, Marcin Grabowski

Data curation: Joanna Michalik, Andrzej Cacko, Jakub Poliński, Kacper Pawlik, Emanuel Tataj

Funding acquisition: Andrzej Cacko, Grzegorz Opolski, Marcin Grabowski

Methodology: Joanna Michalik, Andrzej Cacko, Jakub Poliński, Kacper Pawlik, Emanuel Tataj, Monika Gawałko, Grzegorz Opolski, Marcin Grabowski

Writing – original draft: Joanna Michalik, Andrzej Cacko, Jakub Poliński, Kacper Pawlik, Monika Gawałko, Marcin Grabowski

Writing – review & editing: Joanna Michalik, Andrzej Cacko, Jakub Poliński, Kacper Pawlik, Emanuel Tataj, Monika Gawałko, Grzegorz Opolski, Marcin Grabowski

**Conceptualization:** Joanna Michalik, Andrzej Cacko, Jakub Poliński, Kacper Pawlik, Emanuel Tataj, Monika Gawałko, Grzegorz Opolski, Marcin Grabowski.

**Data curation:** Joanna Michalik, Andrzej Cacko, Jakub Poliński, Kacper Pawlik, Emanuel Tataj.

**Funding acquisition:** Andrzej Cacko, Grzegorz Opolski, Marcin Grabowski.

**Methodology:** Joanna Michalik, Andrzej Cacko, Jakub Poliński, Kacper Pawlik, Emanuel Tataj, Monika Gawałko, Grzegorz Opolski, Marcin Grabowski.

**Writing – original draft:** Joanna Michalik, Andrzej Cacko, Jakub Poliński, Kacper Pawlik, Monika Gawałko, Marcin Grabowski.

**Writing – review & editing:** Joanna Michalik, Andrzej Cacko, Jakub Poliński, Kacper Pawlik, Emanuel Tataj, Monika Gawałko, Grzegorz Opolski, Marcin Grabowski.
